# Lipid-Coated Nanobubbles in Plants

**DOI:** 10.3390/nano13111776

**Published:** 2023-05-31

**Authors:** Stephen Ingram, Steven Jansen, H. Jochen Schenk

**Affiliations:** 1 Institute for Atmospheric and Earth System Research/Physics, University of Helsinki, 00560 Helsinki, Finland; 2 Institute of Botany, Ulm University, 89081 Ulm, Germany; 3 Department of Biological Science, California State University Fullerton, Fullerton, CA 92831-3599, USA

**Keywords:** nanobubbles, polar lipids, plants, xylem, negative pressure

## Abstract

One of the more surprising occurrences of bulk nanobubbles is in the sap inside the vascular transport system of flowering plants, the xylem. In plants, nanobubbles are subjected to negative pressure in the water and to large pressure fluctuations, sometimes encompassing pressure changes of several MPa over the course of a single day, as well as wide temperature fluctuations. Here, we review the evidence for nanobubbles in plants and for polar lipids that coat them, allowing nanobubbles to persist in this dynamic environment. The review addresses how the dynamic surface tension of polar lipid monolayers allows nanobubbles to avoid dissolution or unstable expansion under negative liquid pressure. In addition, we discuss theoretical considerations about the formation of lipid-coated nanobubbles in plants from gas-filled spaces in the xylem and the role of mesoporous fibrous pit membranes between xylem conduits in creating the bubbles, driven by the pressure gradient between the gas and liquid phase. We discuss the role of surface charges in preventing nanobubble coalescence, and conclude by addressing a number of open questions about nanobubbles in plants.

## 1. Introduction

When first proposed [1], the idea that nanobubbles could exist in sap in the vascular systems of plants was quite controversial. There was much debate among scientists as to whether gas nanobubbles could be stable in a bulk solution of water under constant atmospheric pressure, because high internal gas pressure in nanobubbles caused by surface tension would drive the gas into solution and dissolve the bubbles [2,3,4] unless the surrounding water were already super-saturated with gas. Despite this prediction, nanobubbles in aqueous bulk solution under atmospheric pressure have been found to be surprisingly stable [2,5,6,7,8]. There can be different reasons for this stability, including stabilization by electrostatic double layers at the air–water interface [7,9], stabilization involving hydroxyl ions [10] and electrolytes [11], diffusive shielding of bubble clusters in supersaturated water [12], and especially coating of nanobubbles with surfactant shells that reduce surface tension [4,13,14,15]. In all of the studies, stability of nanobubbles in bulk has been reported for water at or above atmospheric pressure. It may be even more surprising that bulk nanobubbles in water are found in the vascular system of plants, specifically the xylem, where they are subject to negative pressure in the water and large pressure fluctuations sometimes encompassing pressure changes of several MPa over the course of a single day. Here, we present the evidence for nanobubbles in plants and address several questions about them: How can nanobubbles be stable in plant sap and how are they stabilized? How do they form? Why would they not dissolve or coalesce?

## 2. What Is the Evidence for Nanobubbles in Plants?

Water in vascular plants, including angiosperms (the flowering plants, which make up 94% of all plant species), is transported in the xylem, a system of hollow dead conduits with rigid cell walls that connect roots, stems, leaves, and reproductive structures. Xylem is most noticeable as wood in woody plants. The water moving through xylem conduits is referred to as xylem sap. Xylem conduits include unicellular tracheids and multicellular vessels, and all conduits are connected to others via bordered pit pairs, where the sap on its way from one conduit to the next is forced to move through nanopores in so-called pit membranes, which largely consist of a mesh of cellulose microfibrils. Pit membranes are described below in the section addressing how nanobubbles form.

**Figure 1:**
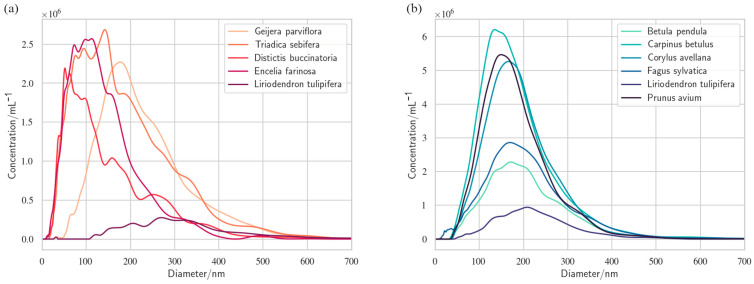
Size distributions and concentrations of nanoparticles observed in eleven flowering plant species. Data taken from (**a**) [18] and (**b**) [19]. Differences in concentrations between the two datasets may be partly caused by the fact that during nanoparticle tracking analysis, the data in (**a**) were collected in moving samples while the data in (**b**) were from stationary samples.

The existence of gas nanobubbles in xylem sap was first hypothesized based on observations of gas flow in wood. When a small hole was drilled into wood and gas flow was measured between the atmosphere and the wood, a net flow of gas into the wood was observed under most circumstances, peaking during the daytime, when sap flow rates are highest. This could not be caused by gas dissolving in sap, because xylem sap is normally gas-saturated or even super-saturated [16], and gas solubility decreases as temperatures increase during the day. Why, then, did gas move into the sap? Some of us (HJS and SJ) hypothesized that the gas moved into sap not by dissolving and not driven by a concentration gradient, but in the shape of gas nanobubbles driven by a pressure gradient [1]. This hypothesis spurred studies of nanobubble concentrations in xylem sap using nanoparticle tracking analysis (NTA) ([17]; Video S1). While NTA does not distinguish between nanobubbles and other nanoparticles, it does allow qualitative assessment of whether the nanoparticles are spherical, which was the case for observations of nanoparticles in all ten flowering plant species whose xylem sap was analyzed using NTA. These included trees, shrubs, and a liana species, all of which contained nanoparticles in their sap ranging between 20 and 300 nm in radius [18,19] (Figure 1).

**Figure 2:**
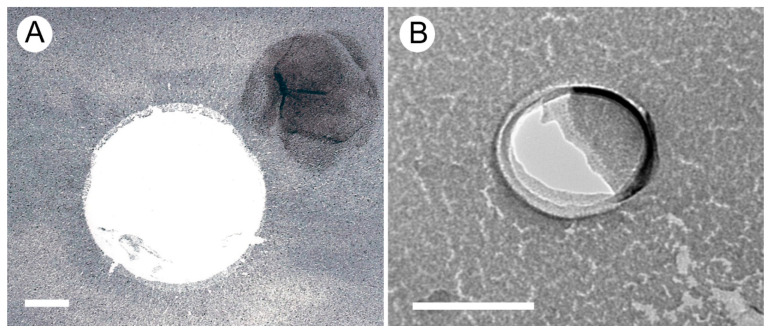
Freeze-fracture EM images of surfactant-coated nanobubbles in *Geijera parviflora* (**A**) and *Corylus avellana* (**B**). The gas bubble cores are visible as the white Pt/C free areas, while the dark Pt/C areas represent the surfactant coat, which can be chopped off, shifted, or wrinkled during the freeze-fracture preparation process. Scale bars = 100 nm.

To assess whether the nanoparticles in xylem sap were indeed nanobubbles, sap from two plant species, *Geijera parviflora* and *Corylus avellana*, was investigated using freeze-facture electron microscopy [18,19], resulting in images such as the one shown in Figure 2, where the spherical bubble shape is clearly visible in the facture plane along with remnants of the surfactant shells that coated the bubble.

## 3. What Is the Evidence for Polar Lipid Coatings of Nanobubbles in Xylem Sap?

**Figure 3:**
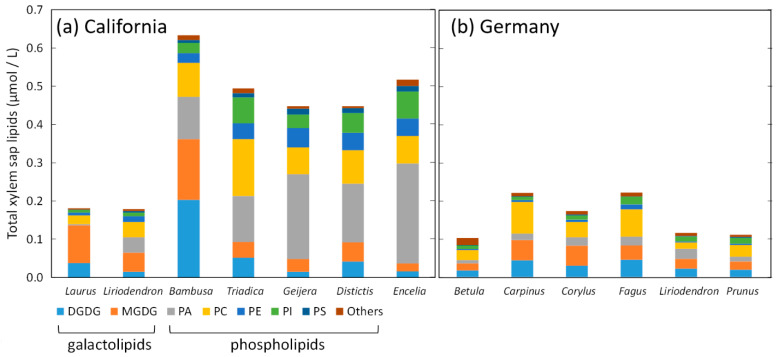
Concentrations and chemical composition of polar lipids in xylem sap in twelve flowering plant species, including data from (**a**) California [21], and (**b**) Germany [19]. DGDG, digalactosyldiacylglycerol; MGDG, monogalactosyldiacylglycerol; PA, phosphatidic acid; PC, phosphatidylcholine; PE, phosphatidylethanolamine; PI, phosphatidylinositol; PS, phosphatidylserine. Figure modified from [21]. See Figure 1 for the full species names.

The discovery that nanobubbles in xylem sap are coated by surfactant shells (Figure 2) was not a surprise, because uncoated nanobubbles would either shrink and dissolve or expand in response to the constant pressure and temperature changes inside a plant. However, this raised the further question of the chemical nature of these shells. Initial analyses of xylem sap residue found evidence both for polar lipids and proteins [18]. The former self-assemble in monolayers at water–gas interfaces, and the latter can be surface-active as well depending on their composition [20]. Polar lipids extracted from xylem sap of thirteen flowering plant species and analyzed via mass spectrometry [19,21] included both phospholipids and galactolipids, with a high proportion of phosphatidic acid (PA), a phospholipid with a negatively charged headgroup. Other charged phospholipids in xylem include negatively charged phosphatidylglycerol (PG), phosphatidylinositol (PI), and phosphatidylserine (PS), as well as zwitterionic phosphatidylcholine (PC) and phosphatidylethanolamine (PE). The amount of neutral galactolipids varied widely between species, making up between 7 and 75% of all polar lipids in xylem sap, and about 30% on average (Figure 3).

Polar lipids in xylem of flowering plants do not appear to change in concentration or composition seasonally [19,21] or over the lifetime of a plant. Comparison with the polar lipid composition of living xylem cells showed that the lipid composition is essentially the same in sap and living cells [21], suggesting that the lipids in dead conduits remain there after the rest of the living cell components have been enzymatically removed during the maturation of the conduit from a living cell into a dead hollow conduit.

It is not possible to separate polar lipids that coat nanobubbles in xylem sap from other polar lipids that coat other surfaces or form micelles in xylem; however, because polar lipids self-assemble at gas–water interfaces, it is clear that any polar lipids found in sap can be part of surfactant shells on gas nanobubbles. A significant correlation was found between the concentrations of nanoparticles and the concentrations of polar lipids in sap for three out of five species studied [19], further suggesting that surfactant shells on nanobubbles are made up largely of polar lipids. Proteins could contribute to these surfactant shells as well, including lipid transfer proteins that have surface-active properties and which are commonly found in xylem sap [22,23,24,25,26,27]. More research is needed to determine which proteins can be found in surfactant shells of xylem nanobubbles.

## 4. How Do Nanobubbles Form in Xylem?

Nanobubbles in xylem sap can only exist under the widely fluctuating pressure and temperature conditions experienced by plants if they are covered by surfactant shells. How do they form, though? As functioning xylem cannot be imaged at the nanoscale with currently available technology, there are no direct observations of nanobubbles forming in plants. There are two possibilities: (1) nanobubbles could form inside lipid micelles or lipid bilayers by diffusion of gas from sap through the lipids into the center of the micelles, which are occupied by the hydrophobic acyl chains of polar lipids and as such are largely hydrophobic and free of water [28]; or (2) gas could move into xylem sap via mass flow from other xylem compartments that contain a gas phase [1].

Option (1), diffusion of gas into the center of lipid micelles or bilayers, would only create a bubble if the resulting gas pressure counteracts the molecular forces that pull the lipids together, including hydrogen bonds between hydrophilic headgroups and relatively weak dispersion forces between the hydrophobic tails. Negative pressure in the surrounding sap could provide the necessary pull. However, molecular dynamics situations have shown that at the spatial and time scale applicable to plants, i.e., the volumes of conduits and the duration of negative pressure conditions, it would take on the order of −10 MPa of negative pressure in the sap to pull micelles or bilayers apart and create a void that could be occupied by gas [28]. Very few plant species ever reach −10 MPa of pressure in their xylem, and it could be that the instability of lipid bilayers and micelles under those conditions is the reason why plants cannot operate at more negative pressures [28]. When lipid bilayers are pulled apart under negative pressure the process is very unlikely to result in nanobubbles, because the energy released during this cavitation event would favor creation of a large cavitation void and thereby create a xylem embolism, i.e., fill the whole conduit with gas, rather than create a stable nanobubble. While it is unlikely that cavitation in lipid bilayers or micelles occurs within the normal pressure range in the xylem of most plants, which ranges between 0 and −5 MPa [29], it is important to realize that the probability of such events increases with the volume of xylem and with the duration and strength of negative pressure [28]. Therefore, xylem embolisms could occasionally form through this process.

**Figure 4:**
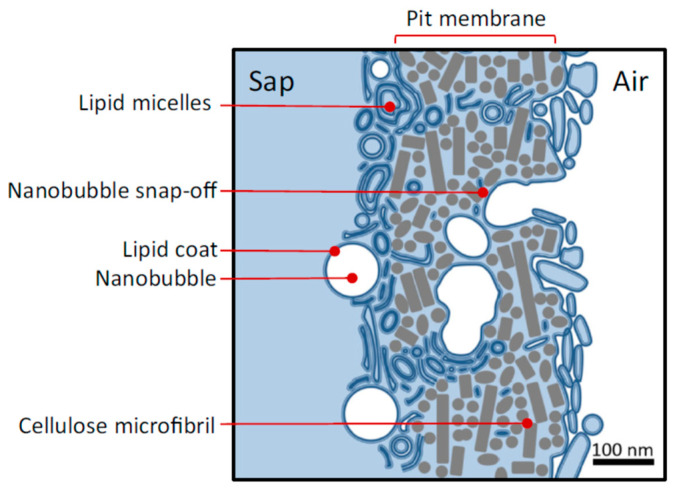
Diagram of nanobubbles forming in a pit membrane of a bordered pit between adjacent conduits. Lipid-coated nanobubbles are formed by a snap-off event due to the presence of surface-active lipids and pore constrictions between cellulose microfibrils. In addition to surfactant-coated nanobubbles, this image shows a wide range of nanoparticle sizes, which can be bilayer to multilayer micelles and vesicles associated with pit membranes. Reprinted with permission from Ref. [21].

Option (2), nanobubble formation from a preexisting gas phase such as an embolized conduit, is far more likely than option (1). Where an embolized (i.e., gas-filled) xylem conduit borders on another one, the gas and liquid phases are separated by mesoporous pit membranes. The discussion here addresses flowering plants, not conifers, which have a very different pit membrane morphology [30]. In flowering plants, these are fibrous membranes ranging in thickness from less than 200 to more than 900 nm [31], largely consisting of aggregated cellulose microfibrils that are between 20 and 30 nm thick [32]. Pores through such fibrous membranes have numerous pore constrictions or pore throats, with thicker membranes having a longer pore pathway and more pore constrictions [32]. The largest constriction size tends to be about 20 nm in diameter [33]. If nanobubbles form in pit membranes between gas-filled and sap-filled conduits, than they would have to form by gas overcoming the surface tension and moving through these constrictions (Figure 4).

According to the Young–Laplace equation, the pressure difference forcing a bubble through a 20 nm pore, assuming a contact angle of zero [34,35] and a pore shape correction factor of 0.5 to account for the fact that pores in fibrous membranes are not cylindrical [1], would be 7.2 MPa, assuming surface tension of pure water of 72 mJ m^−2^. Pit membranes are coated with polar lipids [18,36,37], which reduce the surface tension to about a third of pure water if the polar lipids are in equilibrium [38,39]. At a surface tension of 24 mJ m^−2^, a meniscus could pass through a 20 nm pore with a shape correction factor of 0.5 under a pressure difference between the gas and liquid phase of 2.4 MPa [33]. If pores are slightly larger than 20 nm or surface tension is slightly lower, then the pressure difference required for gas movement through a pore constriction would be even lower. Such pressure differences are well within the range of pressures experienced in the xylem of many plant species [29].

Movement of a meniscus through a pore constriction would not result in a continuous stream of gas from the gas into the liquid phase, instead resulting in snap-off of a nanobubble inside the membrane if the radius of the constriction were less than half the radius of the pore behind it [40,41], which would be the case in fibrous membranes. In geometrically complex pore spaces, the invasion of a non-wetting fluid, such as gas invading a pit membrane wetted with water, occurs not as a simple wetting front, but in rapid snap-off events and so-called Haines jumps [42,43]. Second, the entry of gas into the liquid phase increases the local liquid pressure, which causes bubble snap-off at the pore constriction. Due to the low compressibility of liquid water, the pressure release caused by bubble entry into water under negative pressure is substantial; a bubble that compresses water volume in the confined space within a pit border (around 0.5 to 10 μm^3^ [44]) by only 0.1% releases about 2 MPa of negative pressure [1,45,46]. Third, in liquid that is under negative pressure, minimization of the surface area created by formation of a single small bubble is always thermodynamically favored over rupture of hydrogen bonds between water molecules at the gas–water interface, which requires far more tensile energy density [47]. Therefore, movement of gas through nano-sized pore constrictions under negative pressure initially would produce nanobubbles, not a continuous stream of gas or a cavitation void. Thus, the multiphase interactions between gas, xylem sap, mesoporous pit membranes, and surfactants lead to surfactant-coated nanobubbles.

The pores in pit membranes play another important role for the persistence of nanobubbles in xylem conduits, as they are too small to allow passage of lipid-coated nanoparticles or most lipid micelles (see Figure 1). Therefore, nanobubbles do not move with the sap from one conduit to another or into living cells, and do not accumulate in leaves, instead remaining locked inside individual xylem conduits [19].

The scenario described here for nanobubble creation in pore constrictions depends on the presence of polar lipids inside the pore, lowering the surface tension. Without polar lipids, it would take a far higher pressure difference to force a meniscus through. If polar lipids are present in the pores, then they would invariably coat the nanobubbles because of the strong surface activity of polar lipids. Considering the normal pressures in xylem, the sizes of pore constrictions in pit membranes, and the presence of polar lipids in pit membranes, it appears inevitable that lipid-coated nanobubbles will form in pit membranes that separate gas-filled and sap-filled xylem conduits. The question then arises as to how such bubbles can possibly be stable under the physical conditions that exist in xylem.

## 5. What Physical Conditions Do Nanobubbles Experience in Xylem?

Vascular plants transport sap in xylem, mostly under negative pressure, which is created by evaporation from nanoporous cell walls in leaves, where the liquid phase is in contact with the gas phase inside leaf air spaces. This evaporation is powered by solar energy that causes water vapor from leaves to move into the atmosphere via transpiration through stomata [48]. Evaporation of water vapor from leaf cell walls creates a pull on the liquid phase inside xylem conduits. The upward movement of sap is caused by pulling, through an unbroken chain of water molecules connected to each other via hydrogen bonds in xylem conduits between the roots and leaves. Negative pressure and stretching of water molecules in the sap is caused by this pull from the leaves and by resistances along the pathway from the soil to roots and on to leaves [49]. Changes in the temperature and relative humidity of the atmosphere directly above the leaf cause drastic changes in evaporation rates, and plants respond by opening or closing stomata. At night, transpiration typically declines towards zero, which allows water to move up from the roots into the xylem of stems and leaves and alleviate some of the negative pressure. Depending on the size of the plant and external conditions, diurnal pressure fluctuations can be as large as 2 MPa or even more. Certain plants can create positive pressure in the xylem overnight, causing a diurnal change from positive to negative pressure [50]. Diurnal temperature fluctuations can be large, especially in continental climates where hot days often alternate with cold nights. Sap moving up from the roots may be substantially warmer or colder than the air, creating temperature gradients [51]. Diurnal fluctuations in pressure and temperature affect the solubility of gas [16], which in turn affects the gas pressure inside nanobubbles according to Henry’s law. Moreover, the gas phase in gas-filled conduits and other gas-filled spaces is subjected to pressure fluctuations. While this would typically be close to atmospheric pressure, stems have few or no openings that allow mass flow of gas to the atmosphere [16]. Stems that heat up in the sun can be much hotter than the surrounding air; therefore, the gas phase inside heated stems would be above atmospheric pressure.

Thus, lipid-coated nanobubbles in xylem exist in a physical environment that is mostly under negative liquid pressure, is subjected to large diurnal pressure fluctuations (possibly going into positive pressure at night), and which experiences gas pressure fluctuations and diurnal temperature changes. When sap is extracted from the xylem, the lipid-coated nanobubbles are under atmospheric pressure, which is predicted to compress the lipid layer [38] and minimize bubble sizes. It is impossible to observe nanobubbles under negative pressure in plants; thus, explaining how they can be stable under the changing physical conditions in xylem requires theoretical considerations, which are discussed next.

## 6. How Do Nanobubbles Respond to Changes in Pressure?

**Figure 5:**
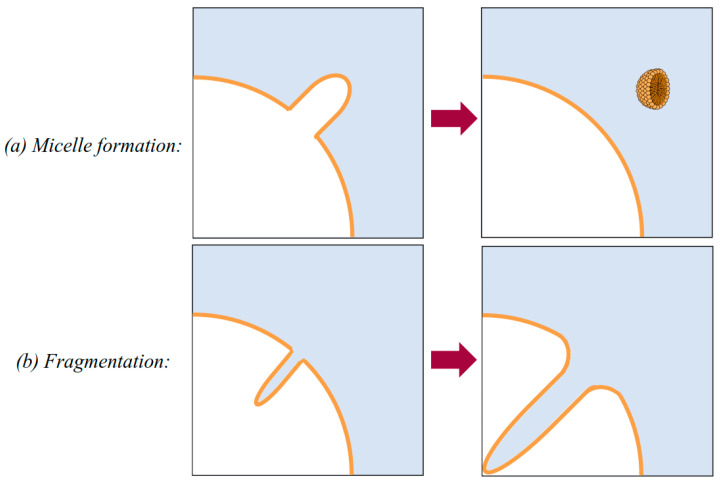
Schematic representations of the buckling of a nanobubble’s coating: (**a**) outward deformation leading to a micelle budding off and (**b**) inward deformation leading to fragmentation of a nanobubble.

Lipid-coated nanobubbles can generally be destabilized by shrinking and dissolution or by expansion into embolism. These correspond to a reduction in radius and an increase in radius, respectively. A rapidly dissolving nanobubble would likely reach a threshold size at which the surface lipids were packed as densely as possible [14]. If the external pressure still exceeded the difference between the internal and Laplace pressures at that moment, i.e., *P_ext_ > P_int_ −* 2γ/r, the interface would buckle and continue receding. If the buckle formed facing inwards, towards the gas, fragmentation into smaller bubbles could then occur. If it buckled outwards, into the water, small groups of lipids could disconnect from the wider bubble surface, diffusing away as micelles or vesicles. Cartoon representations of these phenomena are shown in Figure 5.

If a bubble is at its minimum size as defined by the surface packing, it can continue to shed lipids in order to keep shrinking; however, if it is at its maximum size then it needs to wait until it collides with a micelle or for the tension to increase in order to grow. Therefore, growth induced by more negative external pressure results in a destabilized or potentially ruptured lipid coating. As the area per lipid increases, so does the surface tension; this is sometimes referred to as dynamic surface tension [38]. Lipid coatings are somewhat elastic, as they can undergo phase transitions at critical areas per molecule [52,53] and are able to withstand changes in curvature.

If the larger Laplace pressure implied by the new surface tension remains insufficient to balance the internal and external pressures (2γ/r *< P_ext_ + P_int_*) then bubble expansion into an embolism occurs. In more specific energetic terms, we can say that embolism happens when the unfavorable increase in surface free energy upon rupturing the monolayer (γdA) is outweighed by the favorable change in entropy (*TdS*) upon mechanical expansion of bubble volume *v*, leading to an overall negative change in the Gibbs free energy [54,55,56].

$$dG_{bubble}\left(v\right)=Pdv+\gamma dA\left(v\right)-TdS\left(v\right)<0$$

**Figure 6:**
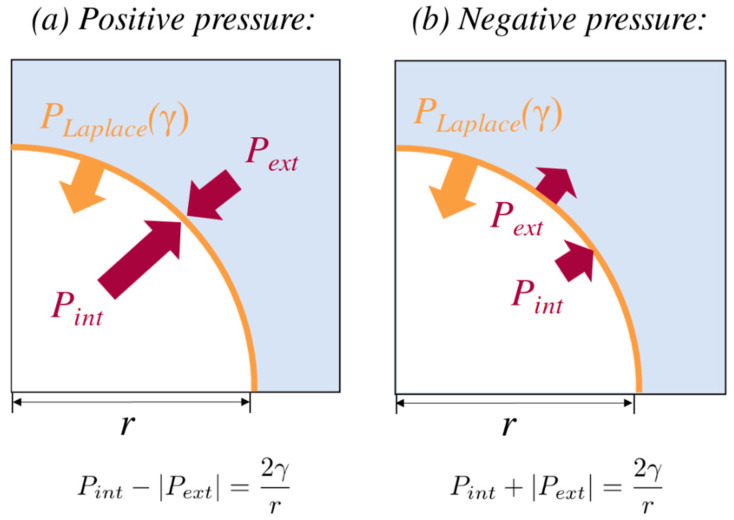
Schematic representation of stable nanobubbles under positive (**a**) and negative (**b**) external pressure, *P_ext_.* Corresponding expressions for the Laplace pressure are shown below the two figures. Note that |*P*| represents the absolute value of the pressure.

The above inequality reveals an important factor in the dynamics of lipid-coated nanobubbles (or bubbles of any size): growth is always favored when the external pressure is negative, and it is only the Laplace pressure of the surface that counteracts the trend towards runaway growth and embolism. The force exerted on the liquid surrounding the coating acts in the same direction as that exerted by the gas present inside the bubble, namely, outwards. The external pressure ‘pulls open’ the interface (see Figure 6).

In contrast, nanobubbles prepared under positive external pressure are believed to be metastable with respect to dissolution [57,58]. Under ambient atmospheric conditions, they exhibit extremely high internal pressures (6 MPa [6], and maybe as high as 14.4 MPa [59]), as they are compressed by the inward force of the liquid.

Removing xylem sap from a plant and exposing it to atmospheric pressure, for instance, by cutting a branch or tapping sap from a tree, flips the microphysics from panel (b) of Figure 6 to panel (a), resulting in an immediate reduction in radius and an increase in *P_int_*. If nanobubbles are present in a natural environment, such as peat and soil, or experience positive pressure within the plant vascular system during the night, they will likely be smaller than when transpirative flux is higher and the surrounding water is stretched.

A note on tension in water at the nanoscale is necessary here. The hydrogen bond network in liquid water is not oriented linearly or vertically; rather, it is stochastic and highly transient, with bonds breaking and forming and neighbors being replaced hundreds of times per nanosecond [60,61].There is, however, an average order on a molecular-length scale; as the neighboring oxygen atoms of a given water molecule arrange themselves into a distorted tetrahedral shape [62,63], the pressure field is isotropic and the nanobubbles are close to spherical in shape.

## 7. How Can Nanobubbles Persist in Bulk Solution without Coalescing?

Another factor affecting nanobubble persistence, in addition to dissolution and expansion into embolism, is coalescence. Why would lipid-coated nanobubbles not coalesce into larger bubbles that would be unstable under negative pressure? The answer lies in surface charges. The so-called Derjaguin, Landau, Verwey, and Overbeek (DLVO) theory [64,65] describes the competition between attractive van der Waals forces and repulsive double-layer forces between nanoscale objects in solution. It states that when the concentrations of salts within the solvent are small, as is the case in xylem sap, electrostatic repulsion dominates the interactions between particles [66]. Nanobubbles have been observed to exhibit negative zeta potentials [67,68], including nanobubbles in xylem sap [18], meaning that they repel each other in solution and coalescence does not occur.

As mentioned above, polar lipids in the xylem of flowering plants include large fractions of negatively charged phospholipids [19,21], including phosphatidic acid, PA, phosphatidylglycerol, PG, phosphatidylinositol, PI, and phosphatidylserine, PS (Figure 3). Depending on lipid composition, neutral lipids may dominate in the xylem of certain plant species; however, negatively charged polar lipids have been found in all species analyzed to date.

We propose that a negative surface charge within bubble coatings can be explained primarily by chemical phenomena. For example, the head group of phosphatidic acid (PA) contains two hydroxyl groups, which have respective pKa values of 3.2 [69] and 11.5 [70]. A pKa is the pH at which 50% of a given proton (H^+^ ion) dissociates into solution; if the external pH is higher than the pKa, more of them dissociate, while if it is lower fewer of them dissociate. Therefore, at the close to neutral pH of xylem sap (6.7), almost every PA molecule is singly deprotonated, producing a surface change density within a given monolayer that is directly proportional to the PA concentration.

In fact, according to data from the CRC handbook of lipid bilayers [71], several of the phospholipids that occur commonly in angiosperm xylem (Figure 3) possess a single negative charge at a physiological pH (Table 1). PC is zwitterionic, meaning that it contains one negative and one positive charge within its head group, as do PE and PS, with the positive centered on the amine groups in all three cases. There may be more finely grained local effects caused by interactions between lipids as well; for instance, Kooijman et al. [69] found that proximity to a PE molecule in a bilayer can induce deprotonation in PA.

Galactolipids contain many proton-donating hydroxyl groups. Using these, the sugar moieties at the water interface can bind directly into the hydrogen bond network, with each oxygen atom fluctuating in charge on a picosecond (10^−12^ s) timescale. This allows the molecule to rapidly conduct electrical energy from the surrounding solvent while maintaining an overall neutral charge [73]. The charge-carrying properties of galactolipids are likely central to their function within chloroplast thylakoid membranes during photosynthesis [74], where they constitute the majority of the membrane [75]. Sulfolipids are negatively charged as well; however, they have been found in very low concentrations in the xylem of flowering plants [21], and are probably not important components of lipid-coated nanobubbles.

Finally, neutral triglycerides (sometimes called triacylglycerides) are found in xylem sap [19,21], and can stably insert themselves into lipid monolayers [76]. While they are neutral molecules, they can each be hydrolyzed into three saturated carboxylic acids by a variety of biochemical and enzymatic processes. These acids likely deprotonate at physiological pH as well.

As a basic example, a bubble with a 50% phospholipid coating at an area of 0.8 nm^2^ per molecule will have an overall charge density of –100 mC/m^2^ (this calculation is independent of radius, and only depends on the area per deprotonated lipid). Charges of this magnitude induce a substantial double-layer force as H^+^ counterions accumulate in the surrounding solvent.

Guan et al. [19] recently observed that 1 mL of xylem sap contains between 5 × 10^8^ and 1.2 × 10^9^ nanoparticles (likely a combination of bubbles, micelles, and solid particles). Were there no electrical forces present, neutral particles would very rapidly coalesce at this concentration due to Brownian motion, producing large aggregates. Videos from nanoparticle tracking analyses of xylem sap (Video S1) and electron microscope studies of frozen xylem sap show mostly individual bubbles (Figure 2), with a few small aggregates observed near pit membranes [18,19], further suggesting that a repulsive force does exist between the nanoparticles. If nanobubbles are unable to pass through pit membranes, and instead slowly accumulate on pit membrane surfaces, they may reduce hydraulic conductivity in the xylem over time.

## 8. How Do Nanobubbles in Xylem Affect Plants?

The cohesion–tension theory [48] does not answer the question of how exactly plants can transport sap under negative pressure in the presence of dissolved gas [16,49]. Lipid-coated nanobubbles may provide a gas reservoir that prevents dissolved gas from coming out of solution and disrupting bonds between water molecules that are necessary for water transport under negative pressure. On the other hand, the lipids may accumulate in pit membranes over time, reduce hydraulic conductance, and perhaps eventually contribute to conduits becoming nonfunctional for water transport. More research is required to determine whether lipid-coated nanobubbles are an essential component of water transport under negative pressure or merely a phenomenon that does not interfere with it.

## 9. Conclusions

There is strong evidence to show that lipid-coated nanobubbles exist in the xylem sap of flowering plants [18,19], including measurements of their sizes and concentrations (Figure 1) and images of their lipid shells (Figure 2). Despite this evidence, currently their existence is not widely acknowledged in plant biology. For example, a recent review of patterns and causes of embolism formation in plants did not mention them even once [77], perhaps because the subject of nanobubbles is outside the expertise of most biologists and requires an interdisciplinary research approach. Certainly, the existence of lipid-coated nanobubbles in the sap of flowering plants subjected to constant changes in pressure and temperature provides a challenge to our understanding both of the physical chemistry of the bubbles and their role in plant water transport. We are only just beginning to address these challenges and various related novel questions [1,18,19,28,38,55]. Much more experimental and theoretical research is required to advance our understanding of how and why these nanobubbles can exist and how they affect the long-distance transport of xylem sap and overall physiology of flowering plants. It remains unclear whether nanobubbles are related to embolism formation in xylem, although they have already resulted in a renewed focus on the gas phase in plants, leading to new approaches to investigating embolism formation. Questions remain about the existence of nanobubbles in gymnosperms, such as conifers, as well as in other vascular plants.

We hope that this review will encourage readers to ask their own questions about nanobubbles in plants and to develop new interdisciplinary approaches to study them, which most likely will require advanced imaging, modeling, and experiments with lipid-coated nanobubbles in artificial systems under negative pressure.

## Figures and Tables

**Table 1:** pKa values for acid and base groups in common plant phospholipids. All values taken from Marsh (2013) [71] except where otherwise referenced. Ammonium pKa values only apply to lipids with ammonium groups (PS, PE, and PC).

Lipid	Phosphate pKa	Ammonium pKa
PS	2.6	11.55
PE	1.7	9.8
PC	1.5	13.9 [72]
PI	2.5	n/a
PA	3.2 [69], 11.5 [70]	n/a
PG	3.5	n/a

## Data Availability

No new data were created or analyzed in this study. Data sharing is not applicable to this article.

## Supplementary Materials

The following supporting information can be downloaded at: https://www.mdpi.com/article/10.3390/nano13111776/s1, Video S1: Video of Nanoparticles (including mainly nanobubbles) in xylem sap freshly extracted from a *Geijera parviflora* tree in Fullerton, California.
